# The Shank3 Interaction Partner ProSAPiP1 Regulates Postsynaptic SPAR Levels and the Maturation of Dendritic Spines in Hippocampal Neurons

**DOI:** 10.3389/fnsyn.2016.00013

**Published:** 2016-05-24

**Authors:** Dominik Reim, Tobias M. Weis, Sonja Halbedl, Jan Philipp Delling, Andreas M. Grabrucker, Tobias M. Boeckers, Michael J. Schmeisser

**Affiliations:** ^1^Institute for Anatomy and Cell Biology, Ulm UniversityUlm, Germany; ^2^International Graduate School in Molecular Medicine, Ulm UniversityUlm, Germany; ^3^WG Molecular Analysis of Synaptopathies, Department of Neurology, Neurocenter of Ulm UniversityUlm, Germany; ^4^Department of Neurology, Ulm UniversityUlm, Germany

**Keywords:** Shank3, ProSAPiP1, PSD-Zip70, LAPSER1, LZTS2, LZTS3, SPAR, synapse

## Abstract

The postsynaptic density or PSD is a submembranous compartment containing a wide array of proteins that contribute to both morphology and function of excitatory glutamatergic synapses. In this study, we have analyzed functional aspects of the Fezzin ProSAP-interacting protein 1 (ProSAPiP1), an interaction partner of the well-known PSD proteins Shank3 and SPAR. Using lentiviral-mediated overexpression and knockdown of ProSAPiP1, we found that this protein is dispensable for the formation of both pre- and postsynaptic specializations *per se*. We further show that ProSAPiP1 regulates SPAR levels at the PSD and the maturation of dendritic spines. In line with previous findings on the ProSAPiP1 homolog PSD-Zip70, we conclude that Fezzins essentially contribute to the maturation of excitatory spine synapses.

## Introduction

The submembranous compartment of excitatory postsynapses contains a large amount of different proteins each contributing to the integrity of the so-called postsynaptic density (PSD; Boeckers, 2006). Among these molecules, the major scaffolding proteins Shank1, Shank2 and Shank3 provide structural and functional stability to the PSD by interconnecting multiple proteins via protein-protein interaction motifs (Sheng and Kim, 2000; Boeckers et al., 2002; Grabrucker et al., 2011a; Sala et al., 2015) i.e., N-terminal Ankyrin repeats, an Scr homology 3 (SH3) domain, a PSD-95/Dlg1/ZO-1 (PDZ) domain, proline-rich clusters and a C-terminal sterile alpha motif (SAM) domain. Intriguingly, mutations in all three human *SHANK* genes have repeatedly been identified in patients with various neuropsychiatric disorders, predominantly autism spectrum disorder (ASD; Leblond et al., 2014). It is therefore crucial to understand the biological functions of both the postsynaptic Shank scaffold and its interacting proteins at the PSD. As the PDZ domain plays a central role in this context, we have yet identified and characterized several binding partners of this protein-protein interaction motif, i.e., the Fezzins ProSAP-interacting protein (ProSAPiP), and LAPSER1 (Wendholt et al., 2006; Schmeisser et al., 2009). The Fezzins comprise four family members, ProSAPiP1, LAPSER1, PSD-Zip70 and N4BP3 that all share a C-terminal Fez1 domain. They further exhibit coiled-coil domains mediating homo- and heteromultimerization among family members and bind to Spine-associated Rap GTPase-activating proteins (SPARs), essential modulators of spine morphology. It is thus hypothesized that Fezzins contribute to synaptic function by interconnecting Shanks and SPARs at the PSD (Maruoka et al., 2005; Wendholt et al., 2006; Spilker et al., 2008; Schmeisser et al., 2009; Mayanagi et al., 2015; Dolnik et al., 2016). However, mechanistic data have only been obtained for PSD-Zip70, which was shown to be critical for mature spine formation and the maintenance of spine maturity involving both SPAR and Rap2 signaling (Maruoka et al., 2005; Mayanagi et al., 2015). Moreover, loss of PSD-Zip70 *in vivo* resulted in increased anxiety and impaired cognition (Mayanagi et al., 2015), behavioral phenotypes remniscient of neuropsychiatric disease. This is indeed of special interest due to the fact that the 8p22 region, which harbors the human *PSD-ZIP70* gene, has been linked to several neuropsychiatric disorders in humans (Tabarés-Seisdedos and Rubenstein, 2009). Interestingly, a study from 2007 further describes an ASD patient with the clinical diagnosis of Asperger’s syndrome and a spontaneous 1.1-Mb deletion of 20p13 encompassing the human ProSAPiP1 gene among others (Sebat et al., 2007). Based on these potentially disease-relevant findings on the Fezzin family and the complete lack of substantial data on the synaptic function of ProSAPiP1, we aimed to analyze this protein in primary hippocampal neurons in more detail. Via ProSAPiP1 overexpression and shRNA-mediated ProSAPiP1 knock-down we show that this molecule is dispensable for the formation of pre- and post synaptic specializations *per se*, but provide evidence that it is involved in the regulation of SPAR levels at the PSD and the maturation of dendritic spines.

## Materials and Methods

### Animal Ethics Statement

All animal experiments in this study were approved by the review board of the Land Baden-Württemberg (Permit Number Nr. O.103) and performed in compliance with the guidelines for the welfare of experimental animals issued by the Federal Government of Germany and the Max Planck Society. Sprague-Dawley rats were purchased from Janvier Labs.

### Antibodies and Vector Constructs

The anti-ProSAPiP1, anti-LAPSER1 and anti-Shank3 antibodies have been described elsewhere (Wendholt et al., 2006; Schmeisser et al., 2009, 2012b). Furthermore, a polyclonal anti-SPAR antibody directed against amino acids 1461–1735 of this protein was generated for this study based on a previously published protocol (Schmeisser et al., 2009). The following antibodies were purchased from commercial suppliers: anti-β-Actin (Sigma-Aldrich) anti-β3-Tubulin (Covance), anti-Bassoon (Enzo Life Sciences), anti-PSD95, anti-VGluT1 and anti-VGAT (all Synaptic Systems). Both the full length ProSAPiP1 cDNA sequence (Wendholt et al., 2006) and RNAi oligonucleotides purchased from Eurofins targeting exon 3 of ProSAPiP1 (5′-GCCTTCAAGCCTGTTGTAC-3′) were cloned into the FUGW vector system. The GFP-SPAR2, GFP-SPAR3 and SPAR-RNAi constructs have been described elsewhere (Richter et al., 2007; Spilker et al., 2008; Dolnik et al., 2016).

### Cell Culture

#### HEK293T Cells

HEK293T cells were kept in DMEM at 37°C in 5% CO_2_ and transfected using polyethylenimine reagent.

#### Primary Neurons

Primary hippocampal neurons were prepared from rat embryos at E18/E19 as described previously (Schmeisser et al., 2013; Halbedl et al., 2016) with minor modifications. In brief, dissected hippocampi were pooled, processed and plated on poly-L-lysine-coated (Sigma-Aldrich) glass coverslips or petri dishes and grown in neurobasal medium complemented with B27 supplement, 0.5 mM L-glutamine and penicillin/streptomycin at 100 U/ml (all reagents from Life Technologies).

#### Viral Infections

Cultured neurons were infected with lentiviruses expressing the following GFP-tagged constructs: scrambled control (Scr), ProSAPiP1-RNAi (RNAi), FUGW empty vector (Vector) or FUGW containing full length ProSAPiP1 (GFP-ProSAPiP1). Viral particles were produced as described previously (Grabrucker et al., 2014). Infection was performed on Day *in vitro* 1 (DIV1) and neurons were processed for either biochemistry or immunolabeling on DIV14 or DIV28, respectively.

The cultured neurons used for spine analysis were additionally infected on DIV24 with RFP-tagged LifeAct lentivirus (Ibidi), visualizing F-actin, and kept in culture until they were processed for microscopy on DIV28.

#### Immunolabeling of Primary Neurons and Fluorescence Imaging

Immunolabeling was performed as described previously (Schmeisser et al., 2012a; Cochoy et al., 2015) with minor modifications. Cells were fixed in 4% paraformaldehyde/4% sucrose, blocked and permeabilized in 2% bovine serum albumin (BSA), 1% horse serum and 0.1% Triton-X-100 and further incubated with primary antibodies. For visualization, secondary antibodies coupled to Alexa Fluor^®^ 488, 568 or 647 (Life Technologies) were used. For fluorescence microscopy, glass coverslips were mounted in VectaMount (Vector labs) containing 4′,6-diamidino-2-phenylindole (DAPI) and images were acquired using an Axioskop 2 and Axiovision Softwares (both from Zeiss).

#### Image Analysis

For quantification of signal number and intensity, the ImageJ Software was used^1^. Puncta were counted along dendrites and puncta density was calculated as puncta per dendrite length. Puncta intensity was measured likewise and shown as relative puncta density normalized to control values.

For the analysis of dendritic spines we deconvolved the RFP signals (F-actin visualized by LifeAct) using the AutoQuant X software (MediaCybernetics). The reconstructed model of dendrites and spines was designed with the Filament Tracer software (Imaris, Bitplane) using default settings. Spines were reconstructed and their length was analyzed with the software. Spine classification was subsequently determined as follows: spines were classified into four categories by the following settings: “Mushroom” (spine length < 2 μm; spine mean width > 0.5 μm; spine neck length > 0.2 μm), “Thin” (spine length < 2 μm; spine mean width < 0.5 μm), “Stubby” (spine length < 2 μm; spine mean width > 0.5 μm; spine neck length < 0.2 μm), and “Filopodia” (spine length > 2 μm; spine mean width < 0.5 μm).

### Protein Biochemistry

#### HEK293T Cell Lysates

HEK293T cells were lyzed in SDS loading buffer (100 mM Tris HCl (pH6.8), 100 mM DTT, 2% SDS, 2 mM EDTA, 20% Glycerol, 0.01% Bromphenol blue (5 mg/ml)) and boiled for 10 min to use for western blot analysis as described below:

#### Subcellular Fractionation

For subcellular fractionation of primary hippocampal neurons, cells were scratched off in phosphate-buffered saline (PBS) containing protease inhibitor mix (Roche), homogenized with a douncer (12 strokes at 900 rpm) and centrifuged at 12,000 × g for 15 min. The pellet containing the crude membrane fraction was resuspended in PBS containing protease inhibitor mix. For obtaining the one-triton extracted PSD fraction, hippocampal tissue from adult rat was fractionated based on a previously published protocol (Distler et al., 2014).

#### Western Blot

Western blotting was performed as previously described (Grabrucker et al., 2011b) with minor modifications. Equal amounts of total protein were separated using SDS-PAGE and blotted on nitrocellulose membranes according to standard protocols. The membranes were further incubated with primary antibodies followed by incubation with HRP-conjugated secondary antibodies. Signals were visualized with ECL Western blotting substrate (Pierce) and the MicroChemi 4.2 machine. For signal quantification, we used the Gelanalyzer Software^2^ and normalized the calculated values against the respective loading controls.

## Results

### ProSAPiP1 Accumulates at Excitatory Synapses in Mature Primary Hippocampal Neurons

**Figure 1:**
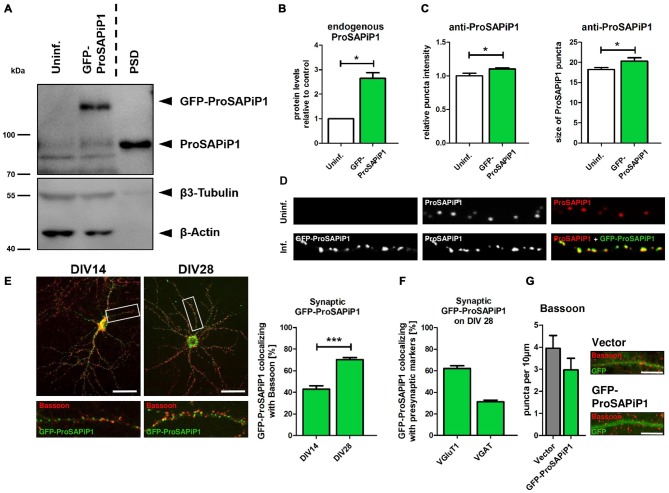
**Synaptic distribution of green fluorescent protein-ProSAP-interacting protein 1 (GFP-ProSAPiP1) in primary hippocampal neurons. (A)** GFP-ProSAPiP1 is clearly visible at the expected molecular weight (~ 125 kDa) in infected primary hippocampal culture at DIV28, whereas no signal is observed in the uninfected control (Uninf.). Post-synaptic density (PSD) fraction from rat hippocampus was loaded as reference for endogenous ProSAPiP1, which is present in all lanes (~ 85 kDa). β3-Tubulin and β-Actin serve as loading control. **(A,B)** Protein levels of endogenous ProSAPiP1 were significantly increased after overexpression of GFP-ProSAPiP1 at DIV28. **(B)** Statistical analysis was performed using unpaired two-sided *t*-test. *p < 0.05; *n* = 3 lysates from three independent cultures for each condition. **(C,D)** Both ProSAPiP1 puncta intensity and ProSAPiP1 cluster size were significantly increased after overexpression of GFP-ProSAPiP1 in primary hippocampal culture at DIV28. Statistical analysis was performed using unpaired two-sided *t*-test. *p < 0.05; *n* = 10 neurons from two independent cultures. **(E)** Exemplary hippocampal neurons, infected with GFP-ProSAPiP1 (green) and immunostained for Bassoon (red) on DIV14 and DIV28 as indicated. Statistical analysis shows a significant increase of synaptic GFP-ProSAPiP1 signals from DIV14 to DIV28 (42.9% on DIV14; 70.3% on DIV28) and was performed using unpaired two-sided *t*-test. ***p < 0.001; *n* = 10 neurons from two independent cultures. **(F)** On DIV28, 62.1% of GFP-ProSAPiP1 signals co-localized with VGluT1 and 31.1% with VGAT, respectively. **(G)** Primary hippocampal cultures were infected with either FUGW empty vector (Vector) or GFP-ProSAPiP1 (both green) and stained for Bassoon (red) on DIV28 as indicated. No significant difference was observed. Scale bar: 10 μm. *n* = 15 neurons from three independent cultures.

To evaluate the subcellular distribution of ProSAPiP1 in hippocampal neurons in more detail, we generated an appropriate cDNA construct and performed lentiviral infection to overexpress GFP-tagged ProSAPiP1. Besides the fact that the Green Fluorescent Protein (GFP) fusion protein was detected at the correct molecular weight (~ 125 kDa) its overexpression also resulted in an increase of endogenous ProSAPiP1 on DIV28 (Figures 1A,B). In line, both intensity and size of the dendritic puncta detected by the anti-ProSAPiP1 antibody were increased and perfectly matched the GFP-ProSAPiP1 signals (Figures 1C,D). Immunostaining of infected cultures on two defined time points of development with anti-Bassoon antibodies further showed that the synaptic distribution of GFP-ProSAPiP1 increases from 42.9% on DIV14 to 70.3% on DIV28 (Figure 1E). This accumulation of GFP-ProSAPiP1 at synapses after 4 weeks in culture mirrors what we have previously shown for the endogenous protein (Wendholt et al., 2006) and strongly supports that ProSAPiP1 might be most relevant for synaptic function at later, more mature stages of neuronal development in culture. Importantly, GFP-ProSAPiP1 is rather found at excitatory (62.1%) than inhibitory (34.1%) contacts on DIV28 (Figure 1F), but does not alter the number of presynaptic specializations in general (Figure 1G).

### ProSAPiP1 is Dispensable for the formation of Presynaptic Specializations in Mature Primary Hippocampal Neurons

**Figure 2:**
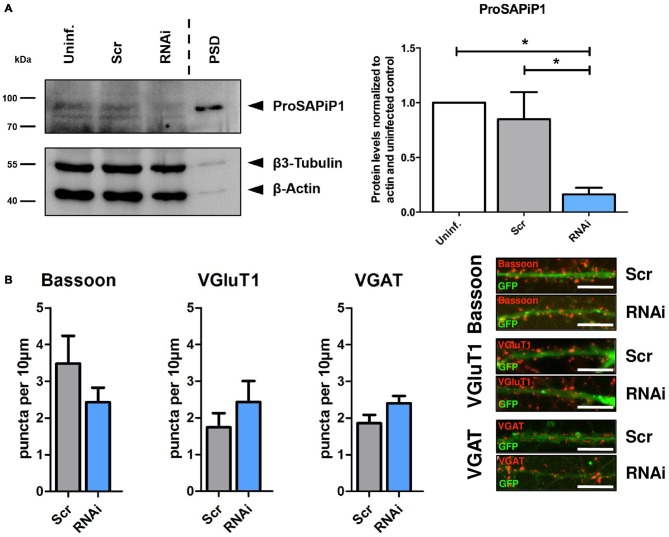
**Analysis of presynaptic specializations after ProSAPiP1 knockdown in mature primary hippocampal neurons. (A)** RNAi based knockdown of ProSAPiP1 in primary hippocampal culture (RNAi) leads to a significant reduction of endogenous ProSAPiP1, whereas there are no differences between the uninfected (Uninf.) and the scrambled control (Scr). PSD from rat hippocampus was loaded as reference for endogenous ProSAPiP1. β3-Tubulin and β-Actin serve as loading control. Statistical analysis was performed using One-way ANOVA. *p < 0.05; *n* = 3 lysates from three independent cultures for each condition. **(B)** Infected (GFP, green) primary hippocampal cultures were stained on DIV28 for Bassoon (red), VGluT1 (red) or VGAT (red) as indicated. No significant differences were observed in the density of Bassoon, VGluT1 or VGAT positive puncta per 10 μm dendrite length between Scr and RNAi. Scale bar: 10 μm. Statistical analysis was performed using unpaired two-sided *t*-test. *n* = 15 neurons from three independent cultures.

We next generated a functional ProSAPiP1 shRNA construct for lentiviral delivery to primary neurons and found a significant downregulation of ProSAPiP1 protein when compared to the appropriate controls (Figure 2A). We further evaluated the effect of a ProSAPiP1 knockdown on the number of presynaptic specializations on DIV28. However, we neither found any change in the number of Bassoon-positive (Figure 2B, left panel) nor in the number of VGluT1-positive excitatory (Figure 2B, center panel) and VGAT-positive inhibitory contacts (Figure 2B, right panel). From these data and our results from Figure 1G, we conclude that ProSAPiP1 is dispensable for the formation of presynaptic specializations in primary hippocampal cultures.

### ProSAPiP1 is Dispensable for Postsynaptic Scaffold Assembly, but Selectively Regulates Postsynaptic SPAR Levels in Mature Primary Hippocampal Neurons

**Figure 3:**
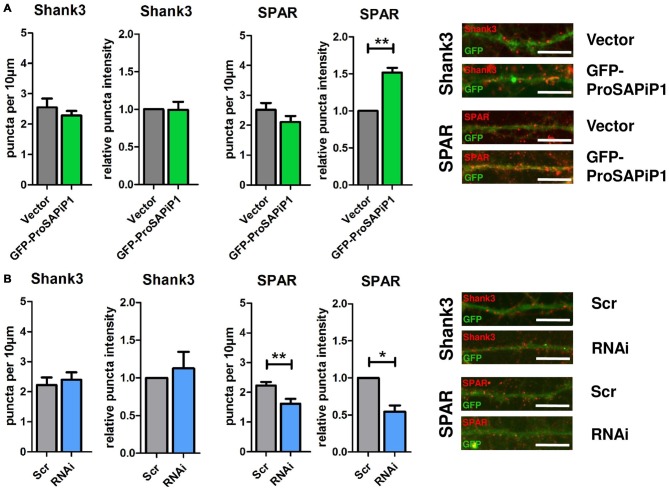
**Analysis of Shank3 and SPAR after ProSAPiP1 overexpression and knockdown in mature primary hippocampal neurons. (A)** Primary hippocampal cultures were infected with either FUGW empty vector (Vector) or GFP-ProSAPiP1 and stained for Shank3 (red) or SPAR (red) on DIV28 as indicated. The intensity of SPAR-positive puncta was significantly increased. **(B)** Primary hippocampal cultures were infected with either Scr or RNAi and stained for Shank3 (red) or SPAR (red) on DIV28 as indicated. A significant reduction was observed in both density and intensity of SPAR positive puncta between Scr and RNAi. Scale bar: 10 μm. Statistical analysis was performed using unpaired two-sided *t*-test. *p < 0.05; **p < 0.01. *n* = 15 neurons from three independent cultures.

We have previously identified two major interaction partners of ProSAPiP1 at the PSD, the key postsynaptic scaffold protein Shank3 and the Spine-associated RapGAP SPAR (Wendholt et al., 2006). We therefore generated a novel polyclonal anti-SPAR antibody (Supplementary Figure 1) and analyzed both density and intensity of synaptic Shank3 and SPAR puncta on DIV28 after ProSAPiP1 overexpression (Figure 3A) and knockdown (Figure 3B). We did not detect any change for Shank3 in either condition (Figures 3A,B, left panels), but found that the intensity of SPAR was increased after ProSAPiP1 overexpression (Figure 3A, right panel) and that both density and intensity of SPAR were decreased after ProSAPiP1 knockdown (Figure 3B, right panel). Further analysis revealed in the same experimental approach that both density and intensity of PSD95—another key postsynaptic scaffold protein and SPAR binding partner (Pak et al., 2001)—were independent from ProSAPiP1 gene dosage (Supplementary Figures 2A,B, left panels). These results implicate that ProSAPiP1 is dispensable for postsynaptic scaffold assembly, but selectively regulates SPAR levels at the PSD. This assumption was further corroborated by the fact that the intensity of LAPSER1—another Fezzin proposed to attach SPAR to the PSD scaffold (Schmeisser et al., 2009)—was selectively increased after ProSAPiP1 overexpression (Supplementary Figures 2A,B, right panels).

### ProSAPiP1 Promotes Dendritic Spine Maturation in Primary Hippocampal Neurons

**Figure 4:**
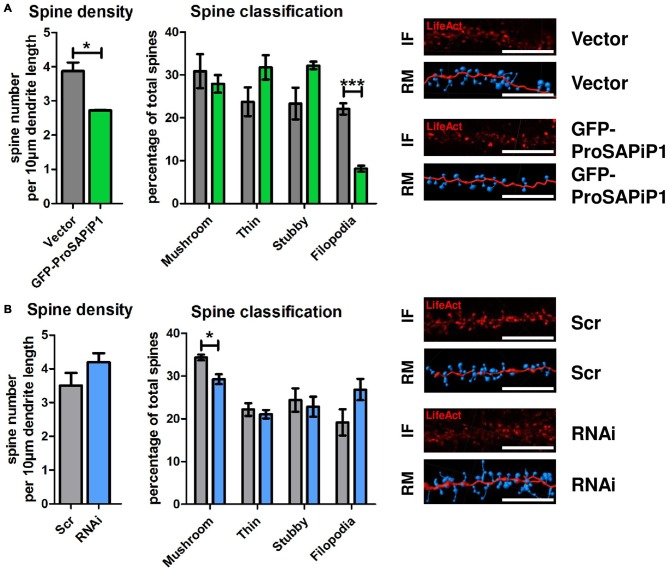
**Analysis of dendritic spines after ProSAPiP1 overexpression and knockdown in mature primary hippocampal neurons. (A,B)** For spine analysis, neurons infected with Vector, GFP-ProSAPiP1, Scr or RNAi were additionally infected with RFP-tagged LifeAct visualizing F-actin on DIV24 and processed for analysis on DIV28. **(A)** Spine density and the percentage of filopodia were significantly decreased in neurons overexpressing GFP-ProSAPiP1 as indicated. **(B)** Spine density remained unchanged between Scr and RNAi while the percentage of mushroom-like spines was significantly decreased after knockdown of ProSAPiP1 as indicated. **(A,B)** IF, immunofluorescence; RM, reconstructed model. Scale bar: 10 μm. Statistical analysis was performed using unpaired two-sided *t*-test. *p < 0.05; ***p < 0.001. *n* = 15 neurons from three independent cultures.

Based on our results and the fact that both PSD-Zip70 and SPAR have repeatedly been associated with modulating the morphology and function of dendritic spines (Pak et al., 2001; Maruoka et al., 2005; Mayanagi et al., 2015), we finally performed an analysis of both spine density and morphology on DIV28 after ProSAPiP1 overexpression (Figure 4A) and knockdown (Figure 4B). We found that overall spine number (Figure 4A, left panel) and the percentage of filopodia (Figure 4A, right panel) were reduced after ProSAPiP1 overexpression. Both effects mirror previously published data on SPAR overexpression (Pak et al., 2001) and are therefore most probably related to the increase of postsynaptic SPAR levels in this experimental condition (Figure 3A, right panel). The effects on spine density and morphology after ProSAPiP1 knockdown were not as strong; we only found a slight, but significant decrease in the number of mushroom-like spines (Figure 4B, right panel). Taken together, these data implicate that a postsynaptic ProSAPiP1/SPAR module promotes the maturation of dendritic spines.

## Discussion

Shank scaffolding proteins are essential for proper synapse function and *SHANK* mutations are associated with various neuropsychiatric disorders (Grabrucker et al., 2011a; Guilmatre et al., 2014). It is therefore of high relevance to understand the molecular interactions of the Shanks in more detail. However, the precise role of several Shank binding partners at the synapse is still unclear and needs to be resolved for a better understanding of Shank synaptic biology and synaptopathic disease alike.

This study was aimed at unraveling functional aspects of the Fezzin family member ProSAPiP1, a postsynaptic protein we originally identified as binding partner of both Shank3 and SPAR (Wendholt et al., 2006). It localizes to synaptic contacts at later stages of synapse maturation and might also be related to neuropsychiatric disease (Wendholt et al., 2006; Sebat et al., 2007). After viral infection of primary hippocampal neurons with an appropriate expression construct, we found that GFP-ProSAPiP1 accumulates predominantly at excitatory synapses at later, mature stages of neuronal development in culture. Interestingly, overexpression of GFP-ProSAPiP1 also resulted in an increase of endogenous ProSAPiP1 implicating the formation of ProSAPiP1 multimers comprising both the endogenous and exogenous protein. Due to the fact that Shank3 is crucial for synapse formation (Roussignol et al., 2005; Grabrucker et al., 2011a; Verpelli et al., 2011; Arons et al., 2012), we further asked if this was—at least in part—depending on its molecular interaction with ProSAPiP1. However, neither an increase nor a decrease of ProSAPiP1 gene dosage did alter the number of presynaptic specializations in primary hippocampal neurons. Interestingly, similar results have been obtained for the other Fezzin family member and ProSAPiP1 homolog PSD-Zip70 with the exception that VGluT1 puncta density was reduced in cortical neurons from PSD-Zip70 KO mice (Maruoka et al., 2005; Mayanagi et al., 2015). Thus, Fezzins like ProSAPiP1 seem to be rather dispensable for synapse formation in culture—contrary to other Shank3 interactors such as Abelson interacting protein 1 (Abi-1; Proepper et al., 2007) or Actin-binding protein 1 (Abp1; Haeckel et al., 2008). In line, we could show that the assembly of key postsynaptic scaffold proteins such as Shank3 or PSD95 is independent of ProSAPiP1 gene dosage as there was no change in both density and intensity of postsynaptic Shank3 or PSD95 after either overexpression or knockdown of ProSAPiP1. However, further analyses revealed that ProSAPiP1 gene dosage indeed has an impact on the levels of its other interaction partner SPAR, a Spine-associated Rap GTPase activating protein, which stabilizes mature spine synapses (Pak et al., 2001; Spilker and Kreutz, 2010; Mihalas et al., 2013). Overexpression of ProSAPiP1 resulted in an increase, ProSAPiP1 knockdown in a decrease of postsynaptic SPAR intensity. The additional decrease of SPAR density after ProSAPiP1 knockdown could be explained by the fact that the loss of ProSAPiP1 leads to a general reduction of Fezzin member heteromers so that SPAR levels are reduced beyond the detection limit at the synapse. Based on previous observations that similar features have been reported for PSD-Zip70 (Maruoka et al., 2005; Mayanagi et al., 2015), it can be hypothesized that Fezzins regulate SPAR levels at the PSD. As SPAR, in turn, is essential for the maturation of dendritic spines (Pak et al., 2001), we finally analyzed the impact of ProSAPiP1 gene dosage on both spine density and morphology. Importantly, we found that overexpression of ProSAPiP1 resulted in a strong reduction of filopodia, while ProSAPiP1 knockdown caused a slight, but significant decrease in mushroom-like spines. These data highly support the notion that ProSAPiP1 is indeed involved in promoting the maturation of dendritic spines—most probably via regulating SPAR levels at the PSD.

Based on our results and given that ProSAPiP1 is a component of the PSD localizing to this subcellular compartment at late stages of neuronal development in culture it can be hypothesized that a ProSAPiP1/SPAR module at the PSD controls synapse maturation and not synaptogenesis *per se*. A recent study in PSD-Zip70 KO mice provided a more detailed investigation of the potential signaling pathways that could be involved. Mayanagi et al. (2015) showed that PSD-Zip70 assists the targeting of SPAR to synapses and thereby modulates Rap2 signaling. The specific role of ProSAPiP1 in this context is still unclear and will be addressed in future investigations. Taken together, our study provides further evidence that Fezzins like ProSAPiP1 fulfill specific molecular functions at the PSD to guarantee the proper maturation of excitatory spine synapses.

## Author Contributions

TMB and MJS designed the research approaches of this study. DR, TMW, SH and JPD carried out experiments; AMG contributed essential reagents. DR and MJS designed all figures and jointly wrote the manuscript with TMB.

## Funding

The research leading to these results has received funding from the Innovative Medicines Initiative Joint Undertaking under grant agreement No. 115300, resources of which are composed of financial contribution from the European Union’s Seventh Framework Programme (FP7/2007–2013) and EFPIA companies’ in kind contribution (EU-AIMS to TMB). AMG is supported by the Else Kröner-Fresenius Stiftung and the Juniorprofessuren-Programm of the State Baden Württemberg. MJS is supported by the Care-for-Rare Foundation and the Eliteprogramm of the Baden-Württemberg Stiftung.

## Conflict of Interest Statement

The authors declare that the research was conducted in the absence of any commercial or financial relationships that could be construed as a potential conflict of interest.

## Footnotes

^1^http://imagej.nih.gov/ij/

^2^http://www.gelanalyzer.com

## Supplementary Material

Supplementary Figure 1 **Characterization of a novel polyclonal anti-SPAR antibody. (A)** Specificity testing of the anti-SPAR antibody. HEK293T cells were co-transfected with GFP-SPAR and either the control vector (Vector) or SPAR RNAi (for characterization of these constructs see Richter et al., 2007). Western blotting of the corresponding cell lysates with anti-SPAR (left panel) and anti-GFP (right panel) showed reduction of GFP-SPAR in the presence of SPAR RNAi. **(B)** HEK293T cells were further transfected with GFP-SPAR, GFP-SPAR2 or GFP-SPAR3. Western blotting of the corresponding cell lysates with anti-SPAR or anti-GFP showed that this antibody is not cross-reactive to SPAR2 and SPAR3.

Supplementary Figure 2 **Analysis of PSD95 and LAPSER1 after ProSAPiP1 overexpression and knockdown in mature primary hippocampal neurons. (A)** Primary hippocampal cultures were infected with either FUGW empty vector (Vector) or GFP-ProSAPiP1 and stained for PSD95 (red) or LAPSER1 (red) on DIV28 as indicated. The intensity of LAPSER1-positive puncta was significantly increased. **(B)** Primary hippocampal cultures were infected with either Scr or RNAi and stained for PSD95 (red) or LAPSER1 (red) on DIV28 as indicated. No significant difference was observed. **(A,B)** Scale bar: 10 μm. Statistical analysis was performed using unpaired two-sided *t*-test. **p* < 0.05. *n* = 15 neurons from three independent cultures.

## Abbreviations

ASD: Autism spectrum disorder
DIV: Days *in vitro*
Fez1: F37/esophageal cancer-related gene coding leucine zipper motif 1
LZTS: Leucine zipper tumor suppressor
ProSAPiP1: ProSAP-interacting protein 1
PDZ: PSD-95/Dlg1/ZO-1
PSD: Postsynaptic density
RapGAP: Rap GTPase-activating protein
RNAi: RNA interference
SAM: Sterile alpha motif
Scr: Scrambled
SH3: Scr homology 3.
