# Supplementary figures and images for: The Shank3 Interaction Partner ProSAPiP1 Regulates Postsynaptic SPAR Levels and the Maturation of Dendritic Spines in Hippocampal Neurons
Source: Front Synaptic Neurosci. 2016 May 24;8:13. doi: 10.3389/fnsyn.2016.00013 (PMC4877498; doi:10.3389/fnsyn.2016.00013)

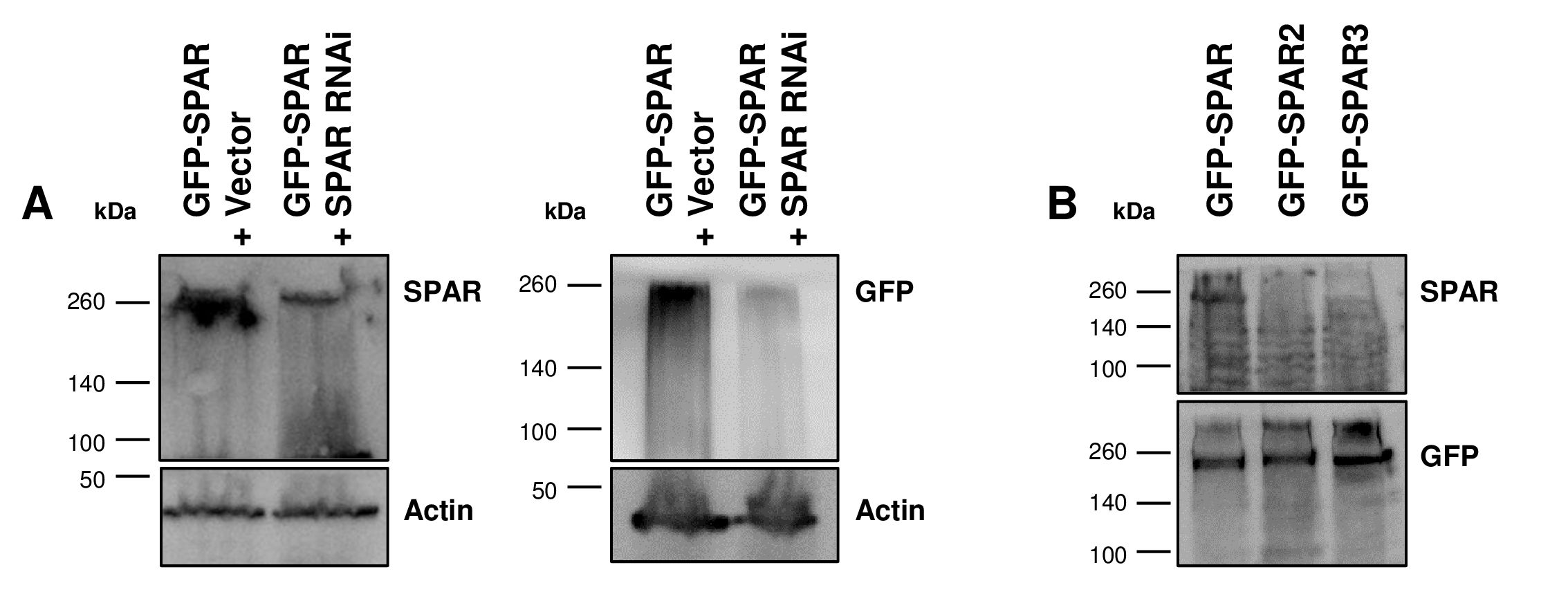

Supplement: Supplementary Figure 1 — Characterization of a novel polyclonal anti-SPAR antibody. (A) Specificity testing of the anti-SPAR antibody. HEK293T cells were co-transfected with GFP-SPAR and either the control vector (Vector) or SPAR RNAi (for characterization of these constructs see Richter et al., 2007). Western blotting of the corresponding cell lysates with anti-SPAR (left panel) and anti-GFP (right panel) showed reduction of GFP-SPAR in the presence of SPAR RNAi. (B) HEK293T cells were further transfected with GFP-SPAR, GFP-SPAR2 or GFP-SPAR3. Western blotting of the corresponding cell lysates with anti-SPAR or anti-GFP showed that this antibody is not cross-reactive to SPAR2 and SPAR3. [file Image_1.jpeg]

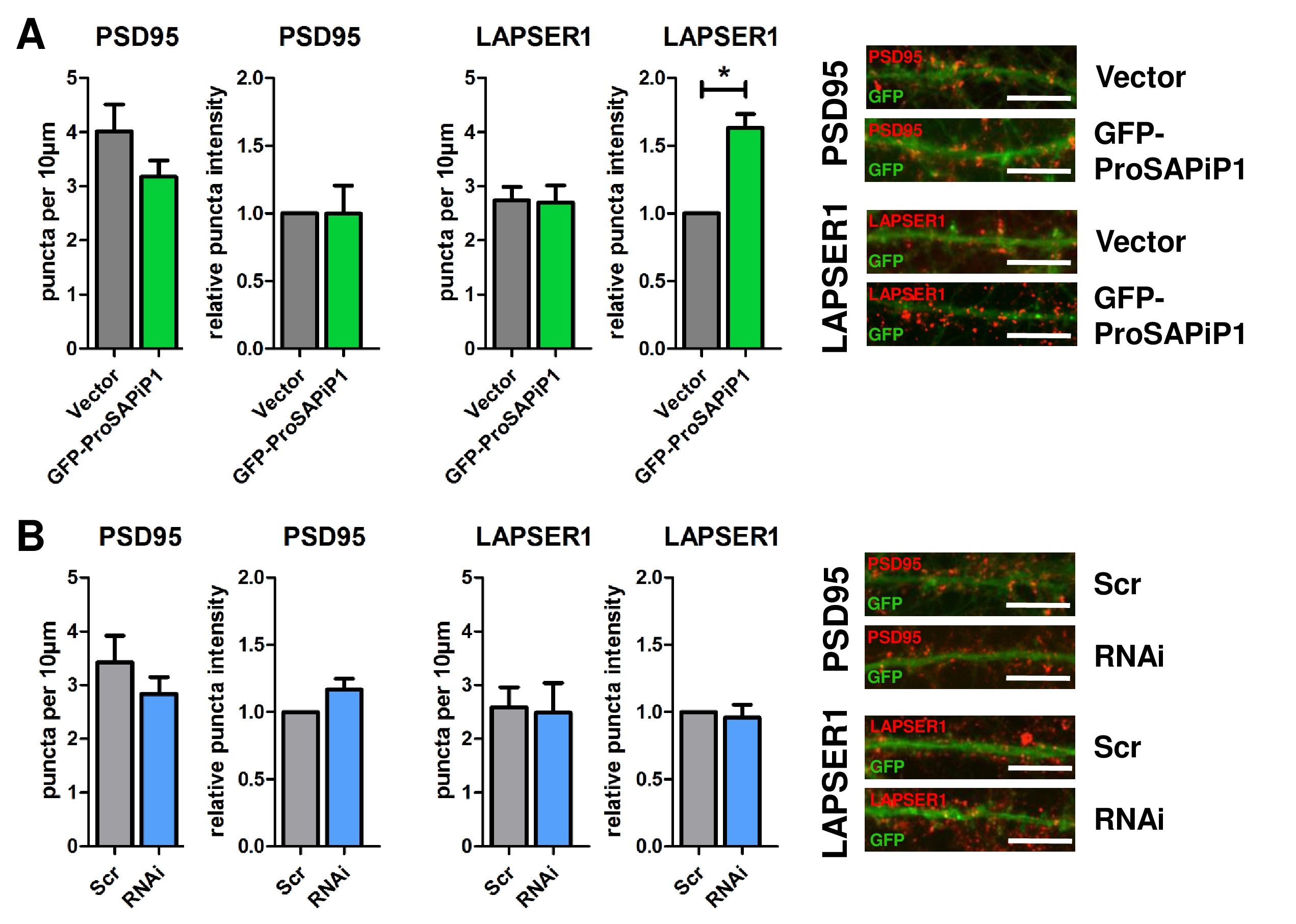

Supplement: Supplementary Figure 2 — Analysis of PSD95 and LAPSER1 after ProSAPiP1 overexpression and knockdown in mature primary hippocampal neurons. (A) Primary hippocampal cultures were infected with either FUGW empty vector (Vector) or GFP-ProSAPiP1 and stained for PSD95 (red) or LAPSER1 (red) on DIV28 as indicated. The intensity of LAPSER1-positive puncta was significantly increased. (B) Primary hippocampal cultures were infected with either Scr or RNAi and stained for PSD95 (red) or LAPSER1 (red) on DIV28 as indicated. No significant difference was observed. (A,B) Scale bar: 10 μm. Statistical analysis was performed using unpaired two-sided t-test. *p < 0.05. n = 15 neurons from three independent cultures. [file Image_2.jpeg]
